# 透明质酸修饰的牛奶外泌体逆转肺腺癌细胞培美曲塞耐药的作用及机制研究

**DOI:** 10.3779/j.issn.1009-3419.2025.102.35

**Published:** 2025-09-20

**Authors:** Lan WU, Jie LEI, Hui LI

**Affiliations:** ^1^010017 呼和浩特，内蒙古医科大学内蒙古临床医学院; ^1^Affiliated Inner Mongolia Clinical College of Inner Mongolia Medical University, Hohehot 010017, China; ^2^014040 包头，内蒙古科技大学包头医学院; ^2^Baotou Medical College, Inner Mongolia University of Science and Technology, Baotou 014040, China

**Keywords:** 肺肿瘤, 外泌体, 透明质酸, 培美曲塞, 耐药, Lung neoplasms, Exosomes, Hyaluronic acid, Pemetrexed, Drug resistance

## Abstract

**背景与目的:**

目前，肺癌发病率及死亡率居全球首位，培美曲塞（Pemetrexed, PMX）是治疗肺腺癌（lung adenocarcinoma, LUAD）的一线药物，但治疗过程中患者易发生耐药。牛奶外泌体（milk exosome, mEXO）具有免疫原性低、组织亲和力高、价格低廉的优势，并且mEXO本身具有抗肿瘤的作用。透明质酸（hyaluronan, HA）是CD44的天然受体，且CD44在LUAD组织中高表达。本文拟构建透明质酸修饰的牛奶外泌体（HA-mEXO），并通过细胞实验初步探讨其逆转PMX耐药性的分子机制。

**方法:**

采用高速离心法从牛奶中提取外泌体，构建HA-mEXO。分别以mEXO、HA-mEXO处理A549、PC-9 PMX耐药细胞株，再采用CCK-8、克隆形成、Transwell及流式细胞术等实验，检测不同处理组耐药细胞的增殖、克隆形成、迁移、侵袭及凋亡表型。最后，通过转录组测序、分析以及细胞功能回复实验，探究HA-mEXO逆转LUAD细胞PMX耐药的机制。

**结果:**

A549、PC-9 LUAD耐药细胞株CD44的表达显著高于亲代细胞，且耐药细胞株对HA-mEXO的摄取率显著高于对mEXO的摄取率。与mEXO组相比，经HA-mEXO处理后的A549、PC-9耐药细胞，PMX半抑制浓度值（half maximal inhibitory concentration, IC_50_）显著下调，克隆形成、迁移、侵袭能力显著降低，而凋亡细胞比例显著上调。Western blot结果显示，与亲代细胞相比，A549、PC-9耐药细胞ZNF516的表达下调，而耐药泵ABCC5表达上调。细胞免疫荧光结果显示，与PBS组相比，HA-mEXO处理组A549、PC-9耐药细胞ABCC5表达下调，而HA-mEXO联合敲减ZNF516处理后的耐药细胞ABCC5的表达却无明显变化。

**结论:**

HA-mEXO携带ZNF516抑制ABCC5表达，从而增强A549、PC-9 LUAD耐药细胞对PMX的敏感性。

## Abstract (en)

**Background and objective:**

Lung cancer currently ranks first globally in both incidence and mortality. Pemetrexed (PMX) serves as a first-line treatment for lung adenocarcinoma (LUAD), but the patients often develop drug resistance during therapy. Milk exosome (mEXO) have the advantages of low immunogenicity, high tissue affinity, and low cost, and mEXO itself has anti-tumor effects. Hyaluronan (HA) naturally bind to CD44, a receptor which is highly expressed in LUAD tissues. This study aims to construct hyaluronan-modified milk exosome (HA-mEXO) and preliminarily investigate their molecular mechanisms for reversing PMX resistance through cellular experiments.

**Methods:**

Exosomes were extracted from milk using high-speed centrifugation, and HA-mEXO was constructed. PMX-resistant A549 and PC-9 cell lines were treated with mEXO and HA-mEXO, respectively. CCK-8 assays, colony formation assays, Transwell assays, and flow cytometry were performed to evaluate proliferation, colony formation, migration, invasion, and apoptosis phenotypes in the treated resistant cell lines. Finally, transcriptomic sequencing, analysis, and cellular functional recovery experiments were conducted to investigate the mechanism by which HA-mEXO reverses PMX resistance in LUAD cells.

**Results:**

The expression of CD44 in A549 and PC-9 LUAD drug-resistant cell lines was significantly higher than that in parental cells, and the uptake rate of HA-mEXO by drug-resistant cell lines was significantly higher than that of mEXO. Compared to the mEXO group, HA-mEXO-treated A549 and PC-9 resistant cells exhibited significantly reduced half maximal inhibitory concentration (IC_50_) values for PMX, markedly diminished clonogenic, migratory, and invasive capabilities, and a significantly increased proportion of apoptotic cells. Western blot analysis revealed that, compared to parental cells, A549 and PC-9 drug-resistant cells exhibited downregulated ZNF516 expression and upregulated ABCC5 expression. Immunofluorescence analysis revealed that HA-mEXO treatment downregulated ABCC5 expression in A549 and PC-9 drug-resistant cells compared to the PBS group, whereas co-treatment with HA-mEXO and ZNF516 knockdown showed no significant change in ABCC5 expression.

**Conclusion:**

HA-mEXO carrying ZNF516 suppress ABCC5 expression, thereby enhancing the sensitivity of A549 and PC-9 LAUD drug-resistant cells to PMX.

根据国际癌症研究机构的最新统计，肺癌是全球最常见的肿瘤，也是死亡率最高的肿瘤之一^[1]^。其中，肺腺癌（lung adenocarcinoma, LUAD）是最常见的组织学亚型，约占所有肺癌病例的50%^[2]^。培美曲塞（Pemetrexed, PMX）作为临床一线用药，广泛用于局部晚期或转移性非鳞非小细胞肺癌的治疗。该药物属于多靶点抗叶酸类细胞毒性制剂，其作用机制主要通过抑制胸苷合成酶、二氢叶酸还原酶以及甘氨酰胺核糖核苷酸甲酰转移酶等，使细胞分裂停滞于S期，从而抑制肿瘤细胞生长^[3]^，但治疗过程中易发生耐药。

外泌体是直径30-150 nm的纳米级囊泡，由细胞分泌，呈现出脂质双分子层结构，参与细胞间通讯、疾病发展及免疫调节等过程，是当前生物医学研究的热点^[4,5]^。外泌体具有安全性高、稳定性好、生物相容性高等特点，可作为药物递送的载体^[6,7]^。2010年，Hata等^[8]^从牛奶中分离出了外泌体（milk exosome, mEXO），其生物相容性、安全性高，可口服给药^[9-11]^。CD44是肿瘤干细胞标志物，表达于胚胎干细胞、未分化的细胞和肿瘤细胞。CD44属于干细胞样跨膜糖蛋白受体，其N端可与透明质酸（hyaluronan, HA）特异性结合^[12,13]^。HA是一种天然多糖，在组织稳态、细胞迁移及肿瘤发展中起重要作用^[14,15]^。由于生物相容性高、免疫原性低，HA被广泛用于构建CD44靶向递药系统^[16]^。

本研究从价廉易获取的牛奶中提取外泌体，并构建透明质酸修饰的牛奶外泌体（HA-mEXO），同时构建PMX耐药的LUAD细胞株。以mEXO联合PMX处理耐药细胞株，再通过CCK-8、克隆形成、Transwell等实验，研究HA-mEXO在逆转LUAD细胞PMX耐药中的作用。而且，在生信分析基础上，初步探究HA-mEXO逆转LUAD细胞PMX耐药的分子机制。

## 1 材料与方法

### 1.1 外泌体提取及鉴定

#### 1.1.1 超速离心法提取牛奶外泌体

从新鲜牛奶（四川新希望乳液有限公司，澳洲荷斯坦牛）中提取外泌体。将牛奶分装至50 mL离心管，10,000* g*离心30 min，去除脂肪球、酪蛋白聚集体及细胞碎片，收集脱脂乳。将脱脂乳转移至超速离心管，使用超速离心机（美国BEKMAN，货号A94469），100,000 *g*离心90 min。收集上层乳清组分，4 ^o^C、135,000* g*再离心70 min，弃上清，获得mEXO沉淀。

#### 1.1.2 构建HA-mEXO

合成1,2-二硬脂酰-SN-甘油-3-磷酰乙醇胺-N-马来酰亚胺-聚乙二醇2000-透明质酸（DSPE-PEG2000-HA）：将HA（450 mg, 15.0 μmol）、N-羟基磺基琥珀酰亚胺（2.30 mg, 20.0 μmol）和1-乙基-3-（3-二甲氨基丙基）碳二亚胺（4.79 mg, 25.0 μmol）溶于蒸馏水，搅拌混合溶液，4^ o^C反应4 h。将上述反应液与DSPE-PEG2000-氨基（41.1 mg, 15.0 μmol）在DMF中混合，室温搅拌12 h。氮气吹干所得粘稠溶液，将粉末溶于水，并通过LH-20层析柱纯化。终产物即DSPE-PEG2000-HA，经干燥后，-20^ o^C保存备用。

mEXO与HA共孵育，按20:1质量比将DSPE-PEG2000-HA与mEXO混合，37^ o^C孵育48 h，HA锚定于mEXO表面。3500 *g*离心10 min，去除未结合的DSPE-PEG2000-HA。收集沉淀，得到HA-mEXO。

#### 1.1.3 外泌体鉴定

mEXO、HA-mEXO使用2%磷钨酸染色，采用透射电子显微镜（日本日立高新技术公司，货号HT7800），在80 kV加速电压下观察并记录其形态特征。mEXO、HA-mEXO用去离子水稀释1000倍。取1 mL均质化样品，注入纳米颗粒追踪分析仪（英国Malvern Panalytical，货号NS300）进行粒径分析。

### 1.2 细胞培养、耐药细胞株构建及慢病毒感染

A549人LUAD细胞（ATCC，美国，货号CCL-185）、PC-9人LUAD细胞（上海镜像绮点细胞技术有限公司，货号iCell-h263）分别采用含10% FBS的F-12K培养基、含10% FBS的RPMI-1640培养基，在5% CO₂、37^ o^C培养箱中培养。

A549细胞加入25 μmol/L PMX持续培养10个月后，采用有限稀释法克隆筛选获得耐药株（A549/PMX）。PC-9细胞加入1.2 μmol/L PMX持续培养6个月后，采用有限稀释法克隆筛选获得耐药株（PC-9/PMX）。

ZNF516短发夹干扰慢病毒（short hairpin RNA, shZNF516）购自上海吉凯基因有限公司，该病毒载体为pLKO.1，使用U6启动子，包含嘌呤霉素抗性基因，shZNF516靶向序列为5’-AGACCTCATGCCGCTAGATTT-3’。以shZNF516感染对数生长期A549/PMX、PC-9/PMX细胞，利用嘌呤霉素抗性（3 μg/mL）筛选稳转株细胞。

### 1.3 Western blot实验

使用RIPA裂解液提取总蛋白，经BCA法进行蛋白定量后，随后通过SDS-PAGE分离蛋白，并转印至PVDF膜。4^ o^C下5%脱脂牛奶封闭1 h，一抗孵育12 h，包括TSG101（美国LifeSpan BioSciences，货号LS-C408134）、CD81（德国Antibodies-online，货号ABIN2789419）、Calnexin（美国Novus Biologicals，货号NB100-1965）、透明质酸（美国Cloud-Clone Corp，货号CPA182GE21）、CD44（美国Thermo Fisher Scientific，货号PA5-21419）、抗ABCC5（美国Invitrogen，货号PA5-102678）、抗ZNF516（美国Abcam，货号ab121486）、GAPDH（美国Abcam，货号ab9485）。辣根过氧化物酶标记山羊抗兔IgG（美国Abcam，货号ab97051）二抗孵育1 h。使用BeyoECL Moon化学发光试剂盒（上海碧云天生物技术有限公司，货号P0018FS）显色。

### 1.4 细胞免疫荧光实验

DiR红色荧光染料标记mEXO、HA-mEXO，观察耐药细胞摄取外泌体情况。将mEXO与10 μmol/L DiR碘化物细胞膜深红荧光探针（上海翌圣生物科技股份有限公司，货号40757ES25）在37^ o^C下反应30 min。使用超滤管在3500 *g*、4^ o^C离心10 min去除游离荧光染料，获得DiR标记mEXO。将DiR-mEXO和DiR-HA-mEXO分别与A549/PMX、PC-9/PMX细胞共孵育4 h，随后细胞经4%多聚甲醛固定，行DAPI核染色后，通过荧光显微镜观察并记录mEXO的细胞摄取情况。

PBS、HA-mEXO、HA-mEXO+shCtrl、HA-mEXO+shZNF516处理组LUAD耐药细胞分别接种到培养皿中，以4%多聚甲醛固定15 min，0.3% Triton X-100透化处理10 min，再以1%牛血清密封1 h。随后，ABCC5抗体（1:250）4^ o^C孵育过夜，第2天，山羊抗兔IgG H&L (1:250，美国Abcam，货号ab150077）室温孵育1 h后，使用含DAPI的抗荧光淬灭封片液封片，在荧光显微镜下完成观察。

### 1.5 CCK-8及克隆形成实验

收集对数生长期A549、PC-9亲本或耐药细胞，离心重悬后，按5×10³个/孔加入96孔板中，待细胞贴壁后，加入不同浓度PMX和/或外泌体处理72 h。然后，每孔加入10 μL CCK-8试剂（上海皓元生物医药科技有限公司，货号HY-K0301），孵育2 h后使用酶标仪检测吸光度以计算半抑制浓度（half maximal inhibitory concentration, IC_50_）。

取对数生长期A549、PC-9耐药细胞按1×10³个/孔接种于6孔板，细胞贴壁后，加入PMX（A549/PMX, 5 μmol/L; PC-9/PMX, 0.25 μmol/L）处理，并按30 μg/mL分别添加mEXO、HA-mEXO。细胞培养14 d后终止培养，弃去培养基，依次使用4%多聚甲醛固定30 min和1%结晶紫染色30 min，最后对直径>1.5 mm的细胞克隆进行计数。

### 1.6 Transwell实验

使用24孔Transwell小室，下室置入含10% FBS的趋化培养基，接种2×10⁴个A549、PC-9耐药细胞（无血清培养基重悬）至上室，5% CO₂、37^ o^C培养箱中孵育24 h。依次进行冰甲醇固定30 min、室温晾干、1%结晶紫染色10 min，最终通过光学显微镜进行观察与计数。

Transwell上室加入0.5 μg/150 μL的基质胶（上海碧云天生物技术股份有限公司，货号C0372），37^ o^C聚合3 h。接种2×10⁴个A549、PC-9耐药细胞（无血清培养基重悬）至上室，孵育16 h。未侵袭细胞经棉签轻柔去除后，小室依次经冰甲醇固定30 min和1%结晶紫染色30 min处理，最终通过光学显微镜完成观察与计数。

### 1.7 流式细胞术

流式细胞术分析mEXO、HA-mEXO处理后耐药细胞的凋亡率。A549与PC-9耐药细胞以1×10⁶个/孔的密度被接种于6孔板，添加不同浓度的HA-mEXO（7.5、15、30 μg/mL）或不添加HA-mEXO。细胞经胰酶消化后收集，缓冲液重悬，随后依次加入5 μL Annexin V-FITC和5 μL PI，通过Annexin V-FITC/PI凋亡检测试剂盒进行染色，室温避光孵育10 min，利用流式细胞仪分析细胞凋亡率。

### 1.8 转录组测序及分析

采用TRIzol试剂提取LUAD（A549、PC-9）亲代细胞以及PMX耐药细胞的总RNA，送转录组测序（华大基因公司）。测序结果采用R Deseq2包差异分析方法获得差异表达基因，筛选条件为*P*<1e-5，*|Log2 FC|*>1。结果以火山图形式呈现。

### 1.9 统计学分析

所有统计分析使用GraphPad Prism 10.3.0软件完成。实验结果以均数±标准差（Mean±SD）表示，并采用*t*检验进行统计学处理，*P*<0.05为差异有统计学意义。

## 2 结果

### 2.1 HA-mEXO物化性质表征

透射电镜显示，mEXO、HA-mEXO均见典型的外泌体杯托状结构，膜结构完整且分散性良好（图1A）；纳米粒径分析仪结果显示，mEXO的粒径范围为70-140 nm，平均粒径为103.8 nm，符合外泌体正常粒径，HA-mEXO的粒径范围增大至80-130 nm，平均粒径为112.5 nm（图1B）。Western blot结果显示，mEXO、HA-mEXO均表达外泌体特征性膜蛋白CD81、TSG101，但不表达负向蛋白Calnexin，而且HA条带仅在HA-mEXO中显示（图1C）。

**图1:**
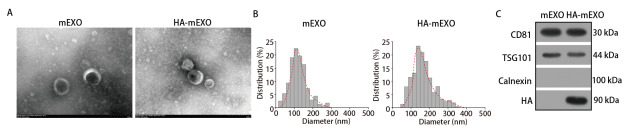
HA-mEXO物化性质表征。A：透射电子显微镜观察mEXO与HA-mEXO的形态特征；B：纳米粒径分析仪评估mEXO与HA-mEXO的粒径大小；C：Western blot验证mEXO与HA-mEXO的特征膜蛋白（CD81和TSG101）、负向蛋白（Calnexin）以及HA的表达。

### 2.2 PMX LUAD耐药细胞株构建

构建了A549、PC-9耐药细胞株，并采用CCK-8检测亲本细胞与耐药细胞对PMX的IC_50_，结果显示，两株耐药细胞IC_50_值均高于亲本细胞（图2A、2B）。Western blot进一步证实耐药细胞中CD44表达上调（图2C）。

**图2:**
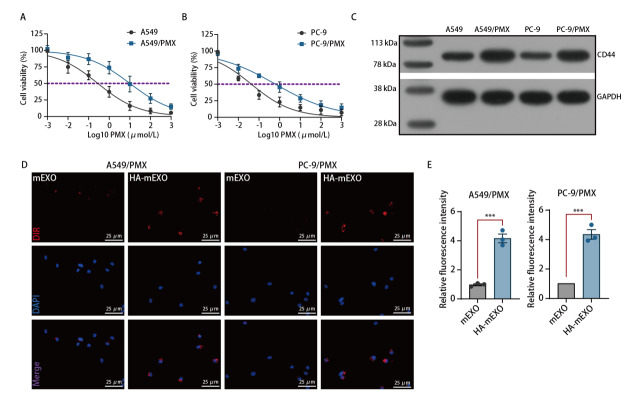
PMX肺腺癌耐药细胞株构建。A、B：CCK-8法验证PMX耐药细胞（A549/PMX与PC-9/PMX）与亲本细胞（A549与PC-9）的IC_50_差异；C：Western blot检测亲本细胞及耐药细胞的CD44膜蛋白表达；D、E：激光共聚焦显微镜观察耐药细胞对mEXO和HA-mEXO的摄取情况。

评估HA修饰是否增加外泌体摄取率，用DiR（红色荧光）标记mEXO、HA-mEXO，分别与A549、PC-9耐药细胞株共孵育。激光共聚焦显微镜显示，两株耐药细胞对HA-mEXO的摄取率均高于mEXO（图2D、2E）。

### 2.3 HA-mEXO增敏PMX抑制LUAD耐药细胞株的增殖和克隆形成能力

CCK-8显示，当外泌体浓度为30 μg/mL时，mEXO、HA-mEXO不抑制A549/PMX、PC-9/PMX细胞的增殖，而当外泌体浓度高于60 μg/mL时，耐药细胞增殖活性被显著抑制，因此，30 μg/mL浓度的mEXO、HA-mEXO被用于后续实验（图3A）。与PBS对照组相比，A549、PC-9耐药细胞株经mEXO、HA-mEXO处理后IC_50_值（PMX）显著下降，且HA-mEXO相较于mEXO下降更明显（图3B）。

**图3:**
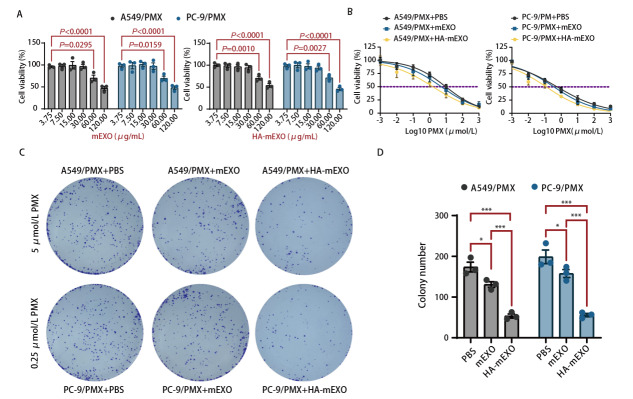
HA-mEXO增敏PMX抑制肺腺癌耐药细胞株的增殖和克隆形成能力。A：不同浓度mEXO、HA-mEXO处理A549、PC-9耐药细胞后的增殖活性；B：CCK-8法检测不同处理组的IC_50_值；C、D：克隆形成实验检测不同处理组细胞克隆形成能力。

耐药细胞株均加入PMX处理，再分别以PBS、mEXO、HA-mEXO处理，克隆形成实验显示，与PBS相比，mEXO、HA-mEXO处理组形成克隆的数目显著下降，能显著下调耐药细胞的菌落数量，而且，在抑制克隆形成方面，HA-mEXO优于mEXO（图3C、3D）。

### 2.4 HA-mEXO增敏PMX抑制LUAD耐药细胞株的迁移及侵袭能力

耐药细胞株均以PMX处理，再分别加入PBS、mEXO、HA-mEXO，Transwell迁移实验显示，与PBS相比，mEXO、HA-mEXO处理组穿梭细胞的数目显著降低，而且，在抑制迁移方面，HA-mEXO优于mEXO（图4A、4B）。Transwell侵袭实验显示，mEXO、HA-mEXO处理组穿梭细胞的数目显著降低，而且，在抑制侵袭方面，HA-mEXO优于mEXO（图4C、4D）。

**图4:**
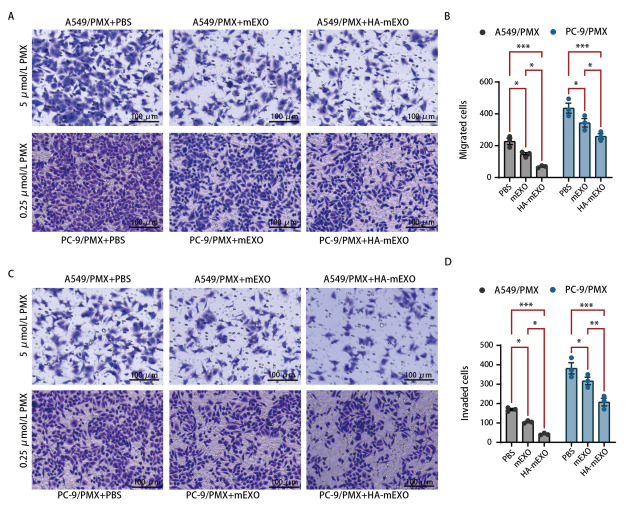
HA-mEXO增敏PMX抑制肺腺癌耐药细胞株的迁移及侵袭能力。A、B：Transwell法检测不同处理组细胞迁移能力；C、D：Transwell法检测不同处理组细胞侵袭能力。

### 2.5 HA-mEXO增敏PMX促进LUAD耐药细胞株的凋亡能力

耐药细胞株均以PMX处理，再分别加入PBS、mEXO、HA-mEXO，收集细胞，染色，经流式细胞检测显示，与PBS组相比，mEXO、HA-mEXO处理组细胞凋亡比例显著增高（图5A、5B），其中HA-mEXO诱导凋亡率高于mEXO。

**图5:**
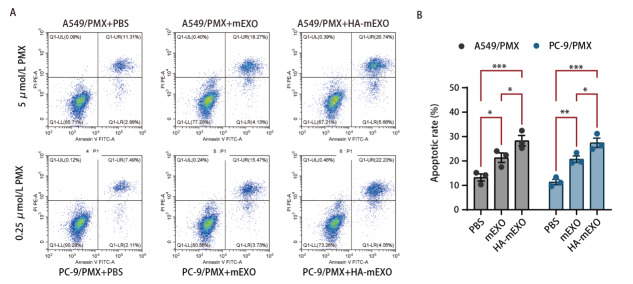
HA-mEXO增敏PMX促进肺腺癌耐药细胞株的凋亡能力。A：流式细胞术检测不同处理组细胞分布图；B：流式细胞术检测不同处理组凋亡细胞比例统计图。

### 2.6 HA-mEXO逆转LUAD细胞PMX耐药机制的初步探索

采用R“DESeq2”包筛选耐药细胞与亲本细胞之间的差异表达基因（图6A），再与mEXO包含的蛋白编码基因取交集，筛选出2个基因：*JMJD1C*和*ZNF516*（图6B）。火山图（图6A）显示，A549耐药细胞株中ZNF516表达下调，而ABCC5表达上调，HA-mEXO可能通过携带ZNF516进而抑制ABCC5表达。Western blot结果显示，与亲本细胞相比，LUAD耐药细胞株中ZNF516表达降低，而ABCC5表达增高（图6C）。为验证ZNF516调控ABCC5的表达，采用PBS、HA-mEXO、HA-mEXO+shCtrl、HA-mEXO+shZNF516处理LUAD耐药细胞株，再检测不同处理组中ABCC5的表达水平，结果显示，与对照组（PBS）相比，HA-mEXO处理组耐药细胞中ABCC5表达显著下调；而与HA-mEXO+shCtrl处理组相比，HA-mEXO+shZNF516处理组耐药细胞中ABCC5表达显著上调（图6D、6E）。

**图6:**
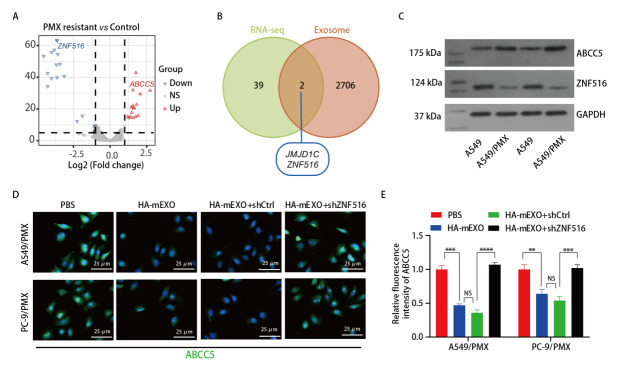
HA-mEXO携带ZNF516通过抑制ABCC5表达逆转肺腺癌细胞PMX耐药。A：肺腺癌耐药细胞和亲本细胞之间基因差异分析火山图（蓝色表示低表达基因，红色表示高表达基因）；B：肺腺癌耐药细胞和亲本细胞之间差异表达基因与mEXO包含蛋白编码基因交集韦恩图；C：肺腺癌耐药细胞和亲本细胞中ZNF516、ABCC5蛋白表达免疫印迹图；D：不同处理组ABCC5表达荧光图；E：不同处理组ABCC5荧光表达差异统计图。

## 3 讨论

PMX是治疗LUAD的一线药物，然而，治疗时其耐药性的产生难以避免，影响治疗效果。鉴于此，探索PMX耐药机制并寻找关键分子，并以此为靶点，抑制PMX外排，提高细胞内PMX浓度，对于增强LUAD细胞对PMX的敏感性具有重要的临床意义。

外泌体是细胞分泌的纳米级脂质双分子层囊泡，通过其腔内包裹的核酸、蛋白、脂质，外泌体参与细胞间物质传递和信息交流。此外，外泌体兼备低免疫原性、高度化学物理稳定性、高组织穿透性能的特点，有望成为药物运载工具^[17]^。与纳米载体相比，mEXO具有靶向递送效果高、与肠上皮细胞亲和力高以及无组织损伤的特点^[18]^。更重要的是，mEXO本身具有抗肿瘤的作用，例如，Martino等^[19]^研究发现mEXO携带miR-27b促进内质网应激介导的结肠癌细胞死亡。本研究证实，低浓度mEXO（30 μg/mL）与PMX联合，可增强A549、PC-9 PMX敏感性，从而增强PMX的抗肿瘤效果，首次证实mEXO具有克服化疗药物耐药的功效，国内外文献尚无报道。

本研究利用LUAD细胞高表达CD44的特性，以CD44天然配体HA修饰mEXO（HA-mEXO），同时构建LUAD耐药细胞株，HA-mEXO可被CD44高表达的耐药细胞靶向摄取。后经验证，HA-mEXO在逆转LUAD细胞PMX耐药、抑制克隆形成、抑制细胞迁移侵袭及促进凋亡方面均显著优于未修饰的mEXO，提示HA-mEXO作为LUAD耐药治疗的潜在优势。目前，由于HA具有无毒、无免疫原性和水溶性好的特点，常被用于治疗CD44过表达的恶性肿瘤^[20]^。Li等^[21]^通过细胞、动物实验结果证实，与未修饰的mEXO负载microRNA-204-5p相比，HA-mEXO抗肿瘤能力显著增强。该研究团队^[22]^利用HA-mEXO负载阿霉素更容易被肺癌细胞靶向摄取，发挥抗肿瘤作用。与上述研究相同，本研究也采用HA修饰mEXO策略以提高外泌体靶向性，然而，我们并未利用HA-mEXO作为抗肿瘤药物或靶向癌基因小干扰RNA的载体，而是研究HA-mEXO本身在逆转LUAD细胞PMX耐药中的作用及机制。

mEXO逆转PMX耐药与其自身携带的功能蛋白有关。通过生信分析，本研究筛选出2个基因：*JMJD1C*和*ZNF516*。文献^[23]^报道，*JMJD1C*是促癌基因，与HA-mEXO逆转LUAD细胞对PMX耐药无关。*ZNF516*通过调控SOX2的转录，抑制结肠癌细胞增殖、侵袭和干细胞样特征，发挥抑制基因的作用^[24]^。所以本研究选择在PMX耐药LUAD细胞中低表达的mEXO特征蛋白ZNF516。ABCC5在肺癌组织中呈高表达，与癌症干细胞的表型相关^[25]^，下调ABCC5的表达可显著降低肿瘤细胞的耐药性^[26]^。生信分析显示，耐药细胞中ZNF516表达显著降低，而ABCC5表达显著升高。Western blot结果亦证实，与亲本细胞相比，PMX耐药LUAD细胞ZNF516表达降低，而ABCC5表达升高。免疫荧光实验证实，以HA-mEXO处理PMX耐药LUAD细胞后，细胞内ABCC5表达显著降低，然而，HA-mEXO联合shZNF526处理PMX耐药细胞，细胞内ABCC5无明显变化，提示HA-mEXO携带的ZNF516经ABCC5抑制LUAD细胞PMX耐药。

本研究以不同浓度梯度mEXO处理LUAD耐药细胞时发现，当浓度大于60 μg/mL时，mEXO具有显著抑瘤效应。在现有基础上，后续我们拟在细胞、动物水平研究mEXO抗肿瘤的作用及机制，具体瘤种包括但不限于LUAD。然而，本研究还存在不足之处，例如并未深入探究ZNF516调控ABCC5表达的分子机制，后续我们拟采用双荧光素酶报告基因实验、染色体免疫共沉淀等实验研究ZNF516是否通过转录调控ABCC5表达，进而导致LUAD细胞对PMX耐药的分子机制。

总之，本研究证实HA可提高mEXO与PMX耐药LUAD细胞之间的亲和性，且HA-mEXO在促进耐药细胞对PMX敏感性、抑制耐药细胞恶性表型方面优于mEXO。机制上，HA-mEXO可能通过携带ZNF516抑制ABCC5的表达进而逆转LUAD细胞对PMX的耐药。
